# Ventricular Tachycardia in Patients With Pre-eclampsia: Prevalence, Predictors, and Associated In-Hospital Adverse Events

**DOI:** 10.7759/cureus.56717

**Published:** 2024-03-22

**Authors:** Omar Elkattawy, Keanaan Malke, David Mothy, Aaron Tran, Sherif Elkattawy, Sayeeda Rab, Ammar Zidat, Omar Mohamed, Fayez Shamoon

**Affiliations:** 1 Internal Medicine, Rutgers University New Jersey Medical School, Newark, USA; 2 Cardiology, St. Joseph's University Medical Center, Paterson, USA; 3 Internal Medicine, Boston Medical Center, Boston, USA; 4 Internal Medicine, Lake Erie College of Osteopathic Medicine, Erie, USA; 5 Medicine, Cooperman Barnabas Medical Center, Livingston, USA

**Keywords:** obstetrics & gynaecology, pre-eclampsia, gestational hypertensive disorder, ventricular tachycardia (vt), cardio-obstetrics

## Abstract

Introduction

Pre-eclampsia is a pregnancy-associated multisystem disorder; in rare cases, it can be complicated by arrhythmias such as ventricular tachycardia (VT). The purpose of this study was to determine the prevalence and predictors of VT among patients admitted with pre-eclampsia as well as to analyze the independent association of VT with in-hospital outcomes in this population.

Methods

Data were obtained from the National Inpatient Sample from January 2016 to December 2019. Patients with a primary diagnosis of pre-eclampsia were selected using International Classification of Diseases, 10th Revision, Clinical Modification (ICD-10-CM) codes. Subsequently, the study population was divided into patients who developed VT versus patients who did not develop this complication. We then assessed the predictors of VT in women with pre-eclampsia as well as the independent association of VT with outcomes taking into account confounders such as age, race, and comorbidities.

Results

Of 255,946 patients with pre-eclampsia, 92 developed VT (0.04%) during their hospital stay. Multivariate logistic regression showed that patients with VT were far more likely to develop cardiac arrest (adjusted odds ratio, or aOR: 92.582, 95% CI: 30.958-276.871, p=0.001), require permanent pacemaker implantation (aOR: 41.866, 95% CI: 14.800-118.432, p=0.001), develop postpartum hemorrhage (aOR: 2.932, 95% CI: 1.655-5.196, p=0.001), and require left heart catheterization (aOR: 19.508, 95% CI: 3.261-116.708, p=0.001). Predictors of VT included being African American (aOR: 1.939, 95% CI: 1.183-3.177, p=0.009), cerebrovascular disease (aOR: 23.109, 95% CI: 6.953-76.802, p=0.001), congestive heart failure (aOR: 50.340, 95% CI: 28.829-87.901, p=0.001), atrial fibrillation (aOR: 20.148, 95% CI: 6.179-65.690, p=0.001), and obstructive sleep apnea, or OSA (aOR: 3.951, 95% CI: 1.486-10.505, p=0.006). Patients in the VT cohort were found to have an increased length of hospital stay compared to the non-VT cohort (7.16 vs. 4.13 days, p=0.001).

Conclusion

In a large cohort of women admitted with pre-eclampsia, we found the prevalence of VT to be <1%. Predictors of VT included conditions such as atrial fibrillation, congestive heart failure, and OSA and being African American. VT was found to be independently associated with several adverse outcomes as well as an increased length of hospital stay.

## Introduction

Pre-eclampsia is a multisystem pregnancy complication that is defined by the onset of gestational hypertension, proteinuria, and the potential to develop eclampsia: a condition that is estimated to cause 10% of maternal mortality in developed nations [1,2]. Given the severity of this pathology, it is critical to study pre-eclampsia, and its interaction with associated conditions; one of these conditions is ventricular tachycardia (VT), a clinically relevant cause of sudden cardiac death [3]. In this study, the relationship between pre-eclampsia and VT is explored through identifying comorbidities that elevate the risk of VT and examining the influence of VT on outcomes among patients with pre-eclampsia.

## Materials and methods

Data acquisition

This is a retrospective database study of the National Inpatient Sample (NIS) database. The NIS is part of the Healthcare Cost and Utilization Project (HCUP) set forth by the Agency for Healthcare Research and Quality. It utilizes International Classification of Diseases, 10th Revision, Clinical Modification (ICD-10-CM) codes for diagnosis and procedures. The data set was utilized to examine data of patients admitted during 2016 to 2019. Encounters with primary diagnosis of pre-eclampsia were selected using ICD-10 code O14. This cohort of patients was further divided into patients who developed VT versus patients without this complication. Adult patients ≥18 years old were included. A total of 255,946 cases met inclusion criteria, comprising 92 patients with pre-eclampsia and VT. An IRB approval was not required as NIS provides de-identified information on patients.

Outcomes and variables

Patient baseline characteristics such as age, race, insurance status were extracted. Comorbidities, hospital complications, mortality rates, disposition status, length of stay, and total charges were also analyzed.

The primary aim of the study was to assess the characteristics and conditions that were predictors of VT in patients admitted with pre-eclampsia. We also assessed whether or not there is a difference in outcomes (mortality, in-hospital complications, length of stay, total charges) between the cohort of patients with pre-eclampsia and VT and patients with pre-eclampsia who did not develop VT. Lastly, we analyzed the independent association of VT with outcomes taking into account confounders such as race, age, and comorbidities.

Statistical analysis

Categorical values were analyzed via Pearson's chi-square analysis and continuous variables were analyzed via independent Student’s t-test. Logistic regression was performed to generate odds ratios (ORs) with 95% confidence intervals (CIs) to assess predictors of VT in women with pre-eclampsia. We also used logistic regression to assess the independent association of VT with outcomes taking into account confounders such as age, race, and comorbidities. A p-value of <0.05 was considered statistically significant. All analyses were completed using IBM SPSS Statistics (IBM Corp., Armonk, NY).

## Results

Of 255,946 patients with pre-eclampsia, 92 developed VT during their hospital stay (0.04%). A statistical analysis of baseline characteristics is summarized in Table 1. Patients who developed VT were less likely to have routine discharges compared to their counterparts (74 (80.4%) vs. 245,265 (95.9%); p=0.001). Similarly, a significant association was found between race and the development of VT, with a larger percentage of patients who identified as African American comprising the VT cohort (34 (37.4%) vs. 56327 (22.8%); p=0.024).

**Table 1:** Baseline characteristics of the study population of pre-eclampsia patients stratified according to developing VT versus not developing VT VT: ventricular tachycardia The data has been represented as n and percentage, or mean ± SD. p-values are significant at <0.05.

Variable		Patients without VT		Patients with VT		Significance (p)
Age (years)		29.4 ± 6.4		30.1 ± 6.4		0.292 (T-value 1.04)
Disposition of the patient	Routine discharge	245,265	95.9%	74	80.4%	0.001
	Transfer to short-term hospital	2589	1.0%	3	3.3%	
	Transfer to other type of facility	383	0.1%	3	3.3%	
	Home health care (HHC)	5413	2.1%	9	9.8%	
	Against medical advice (AMA)	2161	0.8%	2	2.2%	
	Died in the hospital	43	<1%	1	1.1%	
Primary payer	Medicare	2816	1.1%	1	1.1%	0.895
	Medicaid	114,049	44.6%	36	39.6%	
	Private insurance	127,131	49.7%	49	53.8%	
	Self-pay	5169	2.0%	3	3.3%	
	No charge	191	0.1%	0	0.0%	
	Other	6281	2.5%	2	2.2%	
Race	White	116,617	47.2%	32	35.2%	0.024
	African American	56,327	22.8%	34	37.4%	
	Hispanic	50,267	20.4%	17	18.7%	
	Asian or Pacific Islander	11,034	4.5%	3	3.3%	
	Native American	2447	1.0%	0	0.0%	
	Other	10,254	4.2%	5	5.5%	

Univariate analysis results showing the associations between several comorbidities and VT in pre-eclampsia are depicted in Table 2. Univariate analysis showed a higher burden of comorbidities in the VT group including cardiovascular comorbidities such as atrial fibrillation, coronary artery disease, and congestive heart failure. The VT cohort also had a higher incidence of cerebrovascular disease, liver disease and obstructive sleep apnea (OSA).

**Table 2:** Comorbidities for patients who did and did not develop VT during their hospital stay VT: ventricular tachycardia; COPD: chronic obstructive pulmonary disease; HIV: human immunodeficiency virus The data has been represented as n (%). p-values are significant at <0.05.

Variable	Without VT	With VT	p-value
Iron deficiency anemia	6632 (2.6%)	4 (4.3%)	0.289
Congestive heart failure	1128 (0.4%)	22 (23.9%)	0.001
COPD	19,375 (7.6%)	10 (10.9%)	0.232
Coagulopathy	2041 (0.8%)	2 (2.2%)	0.138
Cerebrovascular disease	340 (0.1%)	3 (3.3%)	0.001
Type 2 diabetes mellitus	7207 (2.8%)	3 (3.3%)	0.797
Hypertension	648 (0.3%)	1 (1.1%)	0.112
Alcohol use disorder	355 (0.1%)	0 (0%)	0.721
Liver disease	992 (0.4%)	2 (2.2%)	0.006
Peripheral vascular disease	44 (<1%)	0 (0.0%)	0.900
Atrial fibrillation	169 (0.1%)	4 (4.3%)	0.001
Hypothyroidism	11,828 (4.6%)	2 (2.2%)	0.263
HIV	76 (<1%)	0 (0.0%)	0.869
Coronary artery disease	172 (0.1%)	2 (2.2%)	0.001
Pulmonary hypertension	496 (0.2%)	1 (1.1%)	0.052
Tobacco use disorder	207 (0.1%)	0 (0.0%)	0.785
Obstructive sleep apnea	1434 (0.6%)	5 (5.4%)	0.001
Gestational diabetes mellitus	30,164 (11.8%)	15 (16.3%)	0.179
Cocaine use disorder	870 (0.3%)	1 (1.1%)	0.219
Opioid use disorder	2247 (0.9%)	2 (2.2%)	0.183
Obesity	49,613 (19.4%)	16 (17.4%)	0.628

A summary of crude analysis of outcomes in patients with pre-eclampsia who developed VT versus who did not is included in Table 3. Of patients who developed VT during their hospital stay, 1.1% died compared to <1% who did not have VT (p=0.001). Patients who developed VT had a higher prevalence of complications including cardiac arrest (11 (12%) vs. 93 (<1%); p=.001), shock after delivery (4 (4.3%) vs. 250 (0.1%); p=0.001), non-ST elevated myocardial infarction, or NSTEMI (2 (2.2%) vs. 48(<1%); p=0.001) and postpartum hemorrhage (17 (18.5%) vs. 18,646 (7.3%); p=0.001). They also had higher rates of cardiogenic shock (CS) and required ventilatory support more. The total hospitalization cost was significantly higher in patients with VT compared to those without ($87,394.07 compared to $32,731.48, p=0.001), as was the length of stay (7.16 compared to 4.13 days, p=0.001).

**Table 3:** Outcomes for the study population of pre-eclampsia patients with and without VT VT: ventricular tachycardia; NSTEMI: non-ST elevated myocardial infarction; HELLP: hemolysis, elevated liver enzymes and low platelet count The data has been represented as n (%) or mean ± SD. p-values are significant at <0.05.

Variable	Without VT	With VT	p-value
In-hospital mortality	43 (<1%)	1(1.1%)	0.001
Length of stay (days)	4.13 ± 0.4	7.16 ± 1.03	0.001 (T-value 2.950)
Total hospitalization cost ($)	32731.48 ± 70236	87394.07 ± 11676	0.001 (T-value 4.681)
Cardiac arrest	93 (<1%)	11 (12.0%)	0.001
Permanent pacemaker	214 (0.1%)	6 (6.5%)	0.001
Eclampsia	746 (0.3%)	0 (0.0%)	0.604
HELLP syndrome	10,982 (4.3%)	5 (5.4%)	0.588
Shock after delivery	250(0.1%)	4(4.3%)	0.001
Postpartum hemorrhage	18,646 (7.3%)	17 (18.5%)	0.001
Right heart catheterization	23 (<1%)	2 (2.2%)	0.001
Left heart catheterization	24 (<1%)	4 (4.3%)	0.001
Cardiogenic shock	26 (<1%)	3 (3.3%)	0.001
Mechanical ventilation	274 (0.1%)	4 (4.3%)	0.001
NSTEMI	48 (<1%)	2 (2.2%)	0.001

**Figure 1:**
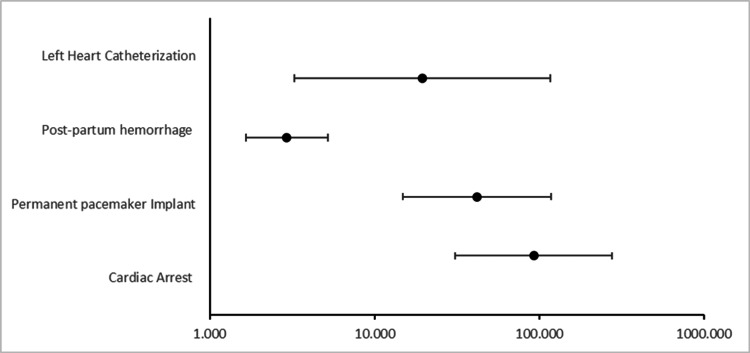
Multivariate logistic regression to assess outcomes in patients who developed ventricular tachycardia

We conducted a multivariate logistic regression to assess the independent association of VT with outcomes taking into account confounders such as age, race, and comorbidities (Figure 1). VT was associated with cardiac arrest (adjusted OR, or aOR: 92.582, 95% CI: 30.958-276.871, p=0.001), permanent pacemaker implantation (aOR: 41.866, 95% CI: 14.800-118.432, p=0.001), postpartum hemorrhage (aOR: 2.932, 95% CI: 1.655-5.196, p=0.001), and left heart catheterization (aOR: 19.508, 95% CI: 3.261-116.708, p=0.001).

**Figure 2:**
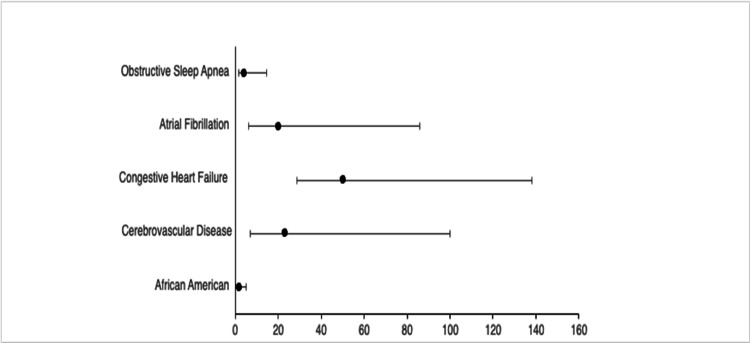
Multivariate logistic regression to assess predictors of ventricular tachycardia in women with pre-eclampsia

We conducted a multivariable logistic regression to assess predictors of VT in patients with pre-eclampsia (Figure 2). VT was significantly associated with cerebrovascular disease (aOR: 23.109, 95% CI: 6.953-76.802, p=0.001), congestive heart failure (aOR: 50.340, 95% CI: 28.829-87.901, p=0.001), atrial fibrillation (aOR: 20.148 95% CI: 6.179-65.690, p=0.001), and OSA (aOR: 3.951, 95% CI: 1.486-10.505, p=0.006). Being African American was also found to be a significant predictor of VT (aOR: 1.939, 95% CI: 1.183-3.177, p=0.009).

## Discussion

Our findings suggest that for patients with pre-eclampsia, the development of VT is associated with an influence on the disposition of the patient, longer hospital stays, and larger hospitalization costs. These results align with previous investigations that found that VT is related to longer hospital stays and larger costs due to VT patients being susceptible to additional procedures [4].

Our findings suggest that the development of VT in patients with pre-eclampsia is associated with a number of characteristics and comorbidities. First, race is suggested to be correlated with the development of VT in pre-eclampsia patients. The current literature supports this claim as young African American adults have been found to show a higher and increasing trend of being hospitalized for VT as compared to White adults from 2005 to 2018 [4]. African American male and female adults also had higher age-adjusted mortality rates in relation to VT between 2007 and 2020 [5]. Among the comorbidities associated with VT, in our univariable analysis, are congestive heart failure, atrial fibrillation, and coronary artery disease. During pregnancy, the cardiovascular system undergoes a number of changes that can promote the development of arrhythmias. These include a decrease in systemic vascular resistance [6,7], expansion of maternal intravascular volume [6], an increase in heart rate, contractility, and cardiac output [7], as well as increased sympathetic activity [8]. Thus, patients with pre-existing cardiovascular-related comorbidities are expected to be at a higher risk of developing arrhythmias including VT during their pregnancy. Our findings are consistent with a prior work that found that patients suffering from congenital heart disease, cardiomyopathy, valvular heart disease, and ischemic heart disease were more likely to develop ventricular tachyarrhythmia during their pregnancy [9]. One study identified the presence of atrial fibrillation to be a strong predictor of adverse outcomes in patients who presented with VT. The rate of VT was found to be higher in atrial fibrillation patients than those without (67% vs. 59%, p=0.001), and these patients were found to have higher mortality rates (36% vs. 18%, p=0.001) [10].

Our univariable analysis was also consistent with previous studies that found that OSA is associated with the development of cardiac disease, including VT. One study that analyzed a large database found a positive association between OSA and VT (p<0.001); however, the odds ratio following multivariate regressions was no longer statistically significant [11]. Another study aimed to determine the relationship between non-sustained ventricular tachycardia (NSVT) and OSA in hypertrophic obstructive cardiomyopathy (HOCM). They concluded that the prevalence of NSVT increased with the severity of OSA [12]. Lastly, we found an association between liver disease and the development of VT in pre-eclampsia patients. With regard to VT, it has previously been found that liver disease is associated with VT in NSTEMI patients and ventricular/supraventricular arrhythmias in patients with diabetes mellitus type 2 (T2DM). One study found the incidence of VT to be 4.5 times higher in patients with non-alcoholic fatty liver disease [13]. Another study found that patients with T2DM who had metabolic dysfunction-associated fatty liver disease had greater rates of paroxysmal supraventricular tachycardia, paroxysmal atrial fibrillation, and combined ventricular tachyarrhythmias [14].

Our findings also suggest that the development of VT in patients with pre-eclampsia is associated with worse outcomes, including significantly higher rates of both cardiac arrest and death during hospitalization. This is unsurprising considering that VT is an established etiology of sudden cardiac death [15]. In addition, our univariate analysis showed that patients with VT experienced significantly higher rates of CS and NSTEMI. This is of great concern, considering that the coincidence of VT and CS or NSTEMI has been shown to be associated with worse patient outcomes. One multicenter study utilizing the FRENSHOCK (French Observatory on the Management of Cardiogenic Shock in 2016) registry determined that patients with CS induced by ventricular arrhythmia (VA) were more likely to require heart transplantation or the implantation of ventricular assist devices within one year following hospitalization than patients with non-VA-related CS, though no difference in mortality was found [16]. Similarly, VT was also found to be a predictor of sudden cardiac death in patients with NSTEMI. An analysis of the MERLIN-TIMI 36 (Metabolic Efficiency With Ranolazine for Less Ischemia in Non-ST-Elevation Acute Coronary Syndrome-Thrombolysis in Myocardial Infarction 36) trial found that patients with non-ST elevation acute coronary syndrome, a broad syndrome that includes NSTEMI and unstable angina, were more likely to suffer from sudden cardiac death if they experienced VT lasting four or more beats [17]. Accordingly, women with pre-eclampsia who develop VT should be monitored with particular care if they are found to develop CS or NSTEMI. Through multivariate analysis, we also found an association between the development of VT and postpartum hemorrhage in pre-eclampsia patients. Previous studies have already determined that pre-eclampsia patients are at a 1.5-times higher risk of developing hemorrhage than patients without hypertensive disorders of pregnancy, and these findings suggest that VT may exacerbate this predisposition [18].

Our study also has some limitations. NIS is an administrative database that uses ICD-10 codes and, thus, is prone to human coding errors. As with observational studies, our results reflect the correlation and cannot infer causation. In addition, long-term outcomes for patients cannot be established because patients were not followed up longitudinally.

## Conclusions

Pre-eclampsia is a hypertensive disorder of pregnancy which, in rare cases, can be complicated by arrhythmias such as VT. To our knowledge, to date, no work has been done to characterize the impact of VT in pre-eclampsia patients. In this study, we aimed to determine which comorbidities and outcomes are significantly associated with VT and pre-eclampsia. As per multivariate regression, pre-eclampsia patients with VT were more likely to have pre-existing obstructive sleep apnea, atrial fibrillation, congestive heart failure, and cerebrovascular disease. These patients were more likely to be African American. Patients with pre-eclampsia who develop VT were more likely to develop cardiac arrest and postpartum hemorrhage compared to their counterparts. These findings may prove useful to clinicians treating pre-eclampsia patients with VT or underlying structural heart defects that may lead to the development of VT.
